# Clinical and Metabolic Predictors of Hypertensive Disorders in Pregnancies Complicated by Gestational Diabetes Mellitus: A Retrospective Cohort Study

**DOI:** 10.3390/jcm15103835

**Published:** 2026-05-15

**Authors:** Laura La Fauci, Rosario D’Anna, Ferdinando Antonio Gulino, Cristina Barracato, Eliana Zangla, Chiara Conti Nibali, Antonino Di Benedetto, Francesco Corrado

**Affiliations:** 1 Department of Human Pathology in Adult and Developmental Age “Gaetano Barresi”, University of Messina, 98125 Messina, Italy; lauralafauci@gmail.com (L.L.F.); barracatocristina@gmail.com (C.B.);; 2 Department of Clinical and Experimental Medicine, University of Messina, 98125 Messina, Italy

**Keywords:** gestational diabetes mellitus, hypertensive disorders, pregnancy

## Abstract

**Introduction:** Hypertensive disorders in pregnancy (HDP) and gestational diabetes mellitus (GDM) represent two significant maternal cardiometabolic disorders closely related to each other. This study aims to identify predictive risk factors for gestational hypertension in patients with GDM within our population. **Methods:** This cohort study was conducted at the Department of Obstetrics and Gynecology, Policlinico “G. Martino” of Messina from January 2012 to December 2019. It included 684 pregnant women diagnosed with GDM by Oral Glucose Tolerance Test (OGTT) according to Italian guidelines. A detailed medical history was taken for each patient to identify potential predictive risk factors for HDP. Patients with pre-existing hypertension or diabetes were excluded. **Results:** Among 684 women with GDM, 69 (10.1%) developed hypertensive disorders of pregnancy (HDP). Women with HDP had a significantly higher pregestational BMI (30.1 ± 7.7 vs. 26.5 ± 5.6 kg/m^2^, *p* = 0.001) and a higher prevalence of obesity (51% vs. 34%, *p* = 0.0001). Post-load glucose at 60 min was higher in the HDP group (178 ± 34 vs. 164 ± 32 mg/dL, *p* = 0.0001), with more women exceeding the diagnostic threshold (>180 mg/dL: 56% vs. 35%, *p* = 0.001). Multivariate analysis confirmed that pregestational obesity and higher 60-min glucose levels during OGTT were the strongest independent predictors of HDP. **Conclusions:** Obesity and glycemia above the cut-off after 1 h during OGTT are predictive risk factors for hypertensive disorders in patients with GDM.

## 1. Introduction

Gestational diabetes mellitus (GDM) and hypertensive disorders of pregnancy (HDP) are among the most common complications affecting pregnant women worldwide, with significant implications for both short- and long-term maternal and neonatal outcomes [1]. HDP are defined and sub-classified into gestational hypertension and preeclampsia according to the American College of Obstetricians and Gynecologists (ACOG) criteria. Gestational hypertension is defined as a systolic blood pressure ≥ 140 mmHg or diastolic blood pressure ≥ 90 mmHg after 20 weeks of gestation in a previously normotensive woman, while preeclampsia is defined as hypertension with the new onset of proteinuria or other signs of end-organ dysfunction [2].

Globally, the prevalence of GDM ranges from 1% to 28%, depending on diagnostic criteria and population studied [3]. Recent evidence indicates an increasing trend in GDM prevalence, partly due to rising obesity rates and changes in diagnostic criteria. Similarly, HDP affects approximately 5–10% of pregnancies and represents a major cause of maternal morbidity and mortality [4].

Recent research suggests a shared pathophysiological basis between GDM and HDP, notably involving chronic low-grade inflammation, endothelial dysfunction, and insulin resistance [5,6]. These factors have been shown to impair vascular homeostasis and contribute to poor placental perfusion, thereby increasing the risk of hypertensive complications and adverse maternal and neonatal outcomes [7]. These systemic alterations are frequently exacerbated by the overproduction of reactive oxygen species, which further impairs the structural integrity of the vascular wall and inhibits the essential physiological adaptations required for a healthy pregnancy progression [8,9].

While many studies have evaluated the individual risk factors for GDM and HDP, fewer have examined predictive metabolic markers within the GDM population that might indicate susceptibility to HDP. Notably, post-load glycemic responses during OGTT may be more predictive of endothelial stress than fasting levels alone [10]. Additionally, prepregnancy obesity has been consistently associated with both GDM and preeclampsia [11,12,13,14].

Additionally, recent studies have also expanded understanding of the molecular and metabolic mechanisms of GDM and its overlap with HDP, highlighting the need for precision medicine approaches [9,15]. Furthermore, machine learning models have shown promise in identifying at-risk pregnancies based on non-invasive biometric and biochemical data.

The novelty of this study lies in its focus on the specific predictive value of the 60-min glucose excursion during the OGTT. While existing literature has established a general association between GDM and HDP, most clinical models focus on pre-existing risk factors such as maternal age and BMI. Our work adds new information by demonstrating that the 1-h post-load glucose value—a metric often disregarded once a GDM diagnosis is confirmed—serves as a potent, independent metabolic ‘stress test’ for the maternal vascular system. By identifying this routinely collected but underutilized parameter as a predictor for HDP, our study provides clinicians with a practical tool for early risk-stratification, without the need for expensive or specialized biomarkers. This specific threshold effectively captures the efficiency of early-phase insulin secretion and peripheral glucose uptake, offering a more precise reflection of the maternal system’s capacity to maintain hemodynamic stability under acute metabolic provocation compared to conventional fasting or late-phase metrics [10,16].

Despite this growing knowledge, few studies have assessed which specific clinical or metabolic factors among patients with GDM predict HDP development. This study aims to address this gap by focusing on all the risk factors outlined in Italian guidelines for the onset of gestational diabetes and the different positive responses to the glucose oral tolerance test as potential early indicators of HDP risk later in pregnancy.

## 2. Materials and Methods

### 2.1. Study Design and Population

This retrospective cohort study was conducted at the Department of Obstetrics and Gynecology, Policlinico “G. Martino” in Messina, Italy, reviewing medical records from January 2012 to December 2019. Patients diagnosed in subsequent years (2020–2022) were excluded due to COVID-related diagnostic disruptions. Inclusion criteria were a confirmed diagnosis of GDM based on the 75 g OGTT according to Italian national guidelines [17].

Women with pre-existing diabetes or hypertension or with incomplete medical records were excluded to isolate the effects of GDM on HDP development. A total of 684 women were included in the final analysis.

### 2.2. Data Collection

Data were extracted from medical records and included maternal age, pregestational BMI, glucose values from OGTT (at 0, 60, and 120 min), prior GDM history, family history of diabetes, and insulin use. Parity was recorded for all participants, distinguishing between primigravidae and multigravidae, as previous obstetric history is a recognized factor influencing the baseline risk for HDP. Patients were categorized based on the number of altered OGTT values (1, 2, or 3) to stratify the severity of glycaemic impairment. The thresholds of 92, 180, and 153 mg/dL were utilized as they represent the standard diagnostic cut-offs for GDM according to Italian national guidelines. Analyzing these specific points allowed us to determine which metabolic excursion—fasting, 1-h, or 2-h post-load—serves as the most sensitive indicator for hypertensive risk.

The primary outcome was the development of HDP, including gestational hypertension and preeclampsia, diagnosed according to ACOG criteria [18,19,20].

Maternal pregestational BMI was calculated as weight in kilograms divided by the square of height in meters (kg/m^2^). Pregestational obesity was explicitly defined as a BMI ≥ 30 kg/m^2^, in accordance with World Health Organization (WHO) classifications.

Missing data were found to be missing at random and occurred in less than 5% of the total dataset. Specifically, missingness ranged from 0% for maternal age to 4.2% for pregestational BMI. These cases were managed using listwise deletion, ensuring that only complete records were utilized for the final multivariate analysis.

### 2.3. Statistical Analysis

Statistical analysis was performed using the statistical package SPSS version 17 for Windows (SPSS Inc., Chicago, IL, USA). Continuous variables were compared using the independent Student’s *t*-test, while categorical variables and proportions were analyzed using the Chi-square test or Fisher’s exact test, as appropriate. For the multivariate logistic regression, variables were initially screened using univariable analysis with a significance threshold of *p* < 0.10 to ensure that potentially important clinical confounders were not prematurely excluded. Subsequently, a stricter threshold of *p* < 0.05 was applied in the final model to identify independent predictors of HDP. This two-step approach ensures that the identified risk factors are not influenced by confounding variables.

To mitigate the risk of overfitting associated with the events-per-variable (EPV) ratio given the 69 observed HDP events, we employed a modeling strategy by selecting predictors through a preliminary univariable screening (*p* < 0.10), and we have provided the full multivariate model output to ensure transparency for all included clinical variables.

### 2.4. Ethical Considerations

The study was conducted in accordance with the Declaration of Helsinki. Informed consent for participation in the study was obtained from all patients prior to enrollment.

## 3. Results

A total of 684 pregnant women with GDM were enrolled in the study; 478 (69.9%) had one altered OGTT value, 137 (20.1%) had two altered values, and 69 (10%) had three altered values. Among them, 69 women (10.1%) developed hypertensive disorders of pregnancy (HDP). The mean maternal age was similar between groups as was the proportion of women older than 35 years. No significant differences were observed for family history of diabetes, previous GDM, previous PCOS, or the need for insulin therapy (Table 1). By contrast, women who developed HDP had a significantly higher pregestational BMI, with a higher prevalence of obesity.

**Figure 1:**
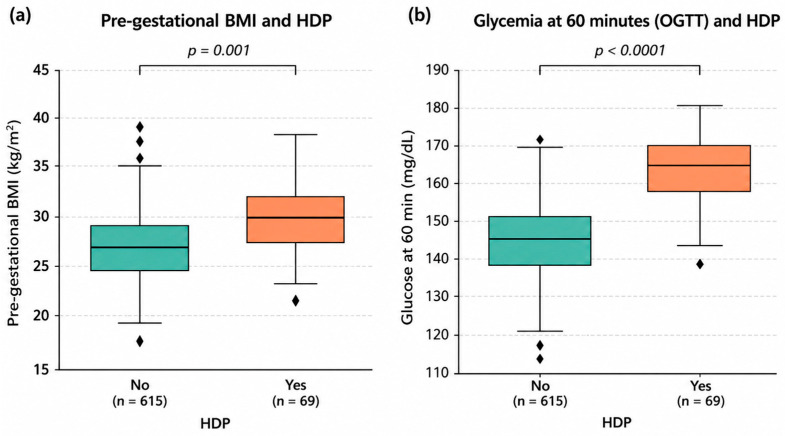
**Clinical and metabolic parameters in women with gestational diabetes mellitus (GDM) stratified by the development of hypertensive disorders of pregnancy (HDP).** (**a**) Boxplot of pre-gestational BMI (kg/m^2^) comparing HDP vs. Non-HDP groups (*p* = 0.001). (**b**) Boxplot of 60-min OGTT glycemia (mg/dL) levels (*p* < 0.001). The central line represents the median, the box indicates the interquartile range (IQR), and whiskers extend to 1.5 times the IQR.

Regarding glucose metabolism, fasting glucose (T0) values were similar, and no difference was observed in the proportion of women with values ≥ 92 mg/dL. Glucose values at 120 min were also comparable, with no difference in the proportion exceeding 153 mg/dL. (Table 1) Conversely, women in the HDP group had significantly higher glucose values at 60 min. (Figure 1) Moreover, the proportion exceeding the diagnostic threshold (≥180 mg/dL) was markedly greater among women with HDP. Figure 1 visually summarizes these differences, highlighting a clear upward shift in both BMI distribution and 60-min glucose values among patients who subsequently developed HDP.

To identify independent predictors of hypertensive disorders of pregnancy (HDP) among women with gestational diabetes mellitus (GDM), a logistic regression analysis was performed. Variables included in the model were selected based on statistical significance in univariate analysis and clinical relevance. These included maternal age, pregestational obesity, prepregnancy BMI, 0, 60 and 120-min OGTT glucose value, insulin therapy, previous GDM and family history of diabetes. Pregestational obesity [OR 3.6 (2.0–6.6) *p* = 0.001], and higher 60-min glucose levels during OGTT [OR 2.4 (1.3–4.2) *p* = 0.02] emerged as the strongest and most consistent independent predictors of HDP.

These findings support the clinical relevance of early metabolic indicators in predicting hypertensive complications in pregnancies complicated by GDM.

## 4. Discussion

This retrospective cohort study, conducted in a real-world Italian tertiary care setting, confirms that pre-pregnancy obesity and post-load hyperglycemia at 60 min during the OGTT are robust and independent predictors of hypertensive disorders of pregnancy in women with gestational diabetes mellitus. The observed HDP incidence of 10.1% aligns with global population-based estimates reporting hypertensive complications in 5–10% of pregnancies, with notably higher rates in high-risk cohorts characterized by obesity or impaired glucose metabolism [2,4,21]. Large-scale systematic reviews have established that GDM is independently associated with an increased risk of preeclampsia, cesarean delivery, and neonatal morbidity, even after adjusting for confounding factors such as Body Mass Index (BMI). This reinforces the paradigm that GDM identifies a specific subgroup of women with an intrinsically elevated obstetric risk profile, likely rooted in underlying genetic and metabolic predispositions [22,23,24].

Our findings underscore the pivotal role of maternal adiposity in the pathophysiology of HDP among women with GDM. Pre-pregnancy obesity emerged as one of the most potent independent predictors in our model. Consistent with current literature, elevated BMI is associated with chronic low-grade inflammation, oxidative stress, and systemic endothelial dysfunction. Specifically, adipose tissue functions as an active endocrine organ, secreting a cascade of pro-inflammatory cytokines and adipokines that can interfere with the physiological remodeling of uterine spiral arteries, thereby contributing to suboptimal placentation and impaired vascular adaptation to the demands of pregnancy [8,9,15,25]. Recent evidence further suggests that increasing BMI in the context of GDM correlates with a risk gradient not only for HDP but also for cesarean section, large-for-gestational-age (LGA) neonates, and Neonatal Intensive Care Unit (NICU) admissions [16,26,27,28,29,30]. These data strongly advocate for the integration of BMI into routine risk stratification algorithms for the GDM population.

A second critical finding is the independent prognostic value of the 60-min glucose value during the diagnostic OGTT. While fasting and 120-min glucose levels did not differ significantly between women with and without HDP, the 1-h levels were markedly higher in those who developed hypertensive complications. This suggests that an exaggerated early post-load glycemic excursion may more accurately capture the synergy of severe insulin resistance and impaired first-phase beta-cell compensation. Whereas fasting glucose primarily reflects hepatic glucose output, the 60-min peak serves as a surrogate for acute endothelial stress and the underlying metabolic instability that precedes vascular collapse [10,16]. By isolating this specific time point, our study moves beyond the binary diagnosis of GDM toward a more granular understanding of which patients are most vulnerable to vascular dysfunction. This observation may also reflect an impaired adaptive cardiovascular response to acute metabolic stress. A rapid glucose excursion during the first hour of the OGTT could contribute to transient endothelial activation, oxidative imbalance, and altered vascular reactivity, mechanisms already implicated in the pathogenesis of hypertensive disorders of pregnancy.

The coexistence of GDM and HDP identifies a particularly high-risk obstetric phenotype. Recent large-scale studies confirm that the combined presence of these disorders is associated with significantly worse maternal and neonatal outcomes than either condition alone, including higher rates of preterm birth and perinatal mortality [26,31,32,33]. Beyond the immediate puerperium, this comorbidity carries profound implications for long-term cardiometabolic health. Pregnancy acts as a natural “stress test”; a failure to maintain metabolic and hemodynamic homeostasis during this period often heralds a systemic fragility that manifests in later decades. Women with a history of both GDM and HDP face an exponentially higher lifetime risk of developing type 2 diabetes, chronic hypertension, myocardial infarction, and stroke [26,27,31,34,35].

The clinical utility of adopting a composite HDP outcome lies in its ability to capture the full spectrum of maternal endothelial stress. Since gestational hypertension and preeclampsia share a common pathophysiological pathway and entail similar vascular risks, evaluating them together provides a more comprehensive indicator of overall hemodynamic instability.

From a pragmatic clinical perspective, utilizing a composite HDP outcome streamlines risk stratification and early intervention. Rather than fragmenting care based on specific hypertensive subtypes, this unified approach justifies the timely initiation of broad preventive strategies, such as low-dose aspirin prophylaxis and intensified maternal–fetal monitoring, thereby optimizing resource allocation and potentially averting severe morbidity.

These results provide actionable insights for precision obstetric care. Simple clinical markers—pre-pregnancy BMI and the 1-h OGTT glucose value—can refine the identification of women who would benefit most from intensified surveillance, aspirin prophylaxis, or more aggressive metabolic management. Although our study is limited by its retrospective, single-center design and the absence of novel biomarkers like angiogenic factors, the use of standardized diagnostic criteria and comprehensive clinical records strengthens the internal validity of the findings. In conclusion, emphasizing these routinely measured parameters allows for a transition from standardized protocols to personalized management, potentially improving both immediate pregnancy outcomes and the long-term health trajectory of the mother and offspring.

Ultimately, the major clinical novelty of our findings lies in repurposing the 60-min OGTT glucose value—which is traditionally disregarded once a GDM diagnosis is established—as a cost-effective, zero-cost metabolic ‘stress test’ that is immediately available to clinicians for early vascular risk stratification.

## 5. Conclusions

In conclusion, our study underscores a crucial and novel clinical paradigm: the 60-min glucose excursion during a routine OGTT is not just a diagnostic threshold for diabetes, but a potent, readily applicable early predictor of hypertensive risk. Integrating this specific, routinely collected parameter into standard protocols allows for an immediate shift toward personalized prenatal care and targeted preventative interventions without adding financial burden to healthcare systems. Because the 60-min OGTT value is already routinely collected in standard prenatal screening, its integration into risk stratification models could be implemented immediately in everyday clinical practice without requiring additional laboratory testing or specialized resources.

Crucially, the immediate clinical applicability of the 60-min OGTT glucose value lies in its dual function. It should no longer be viewed merely as a transient diagnostic threshold for GDM, but rather as an accessible, zero-cost biomarker. Recognizing this early predictor allows clinicians to automatically triage these women into a higher vascular risk tier, establishing a clear pathway for intensified obstetric interventions and targeted long-term cardiometabolic follow-up. Moving forward, combining clinical metrics with advanced technologies, including machine learning and precision nutrition, represents a critical frontier in optimizing maternal–neonatal outcomes and mitigating the lifelong cardiometabolic burden for both the mother and the offspring.

## Figures and Tables

**Table 1:** Baseline characteristics of the study population. Continuous data are expressed as mean ± SD and compared via independent *t*-test. Categorical data are expressed as n (%) and compared via Chi-square or Fisher’s exact test. Significance: *p* < 0.05.

	No HDP	YES HDP	*p*-Value
Parameter	615	69	
Mean maternal age	33.6 ± 5.4	34.5 ± 5.2	0.2
>35 years old	309 (49%)	39 (56%)	0.2
Pregestational BMI	26.5 ± 5.6	30.1 ± 7.7	**0.001**
Pregestational obesity	144 (34%)	35 (51%)	**0.0001**
Family history of diabetes	281 (45.7%)	37 (53.6%)	0.2
Previous GDM	22 (3.6%)	4 (5.8%)	0.3
Previous PCOS	3 (0.4%)	1 (1.4%)	0.8
Insulin therapy required	171 (27%)	19 (27%)	0.9
75 g OGTT Glucose Values			
Mean value T0	94 ± 10	95 ± 9.8	0.4
Mean value T60	164 ± 32	178 ± 34	**0.0001**
Mean value T120	133 ± 34	139 ± 30	0.1
Diagnostic Thresholds			
Values ≥ 92 mg/dL at T0 (%)	469 (74%)	39 (69%)	0.3
Values ≥ 180 mg/dL at T60 (%)	218 (35%)	39 (56%)	**0.001**
Values ≥ 153 mg/dL at T120 (%)	160 (26%)	20 (29%)	0.5

## Data Availability

The data presented in this study are available on reasonable request from the corresponding author. The data are not publicly available due to privacy and ethical restrictions.

## Abbreviations

The following abbreviations are used in this manuscript:

HDP	Hypertensive Disorders in Pregnancy
GDM	Gestational Diabetes Mellitus
OGTT	Oral Glucose Tolerance Test
